# Taste aversion as an under-recognized determinant of bowel preparation quality

**DOI:** 10.3389/fmed.2026.1803366

**Published:** 2026-05-07

**Authors:** Mohammed Abdulrasak

**Affiliations:** 1 Department of Clinical Sciences, Lund University, Malmö, Sweden; 2 Department of Gastroenterology and Nutrition, Skåne University Hospital, Malmö, Sweden

**Keywords:** bedside observation, bowel preparation, colonoscopy quality, taste aversion, topical lidocaine

## Abstract

Inadequate bowel preparation remains a major barrier to high-quality colonoscopy, with taste aversion representing a common yet under-recognized contributor to poor tolerability of the bowel preparation. Current strategies primarily aim to improve palatability through formulation changes or dosing regimens. This paper proposes a novel, hypothesis-driven approach: transient pharmacologic suppression of gustatory perception using topical oral lidocaine to reduce the perceived unpleasantness of bowel preparation solutions. The concept arises from a serendipitous clinical observation in which tongue-focused lidocaine application enabled successful oral intake in a patient with repeated prior intolerance. By targeting taste perception directly rather than modifying the preparation itself, this approach may offer a complementary strategy for selected patients. Further evaluation is required to assess feasibility, safety, and clinical effectiveness.

## Introduction

Inadequate bowel preparation remains one of the most persistent barriers to high-quality colonoscopy (1). Despite decades of refinement in laxative formulations and dosing schedules, up to one in four examinations are compromised by poor bowel preparation (2). Recent population-based data further underscore the magnitude of this problem and the need for additional, patient-centered strategies to improve preparation quality (3). Aversion to the taste of bowel preparation solutions represents one such contributor, often leading to incomplete ingestion, inpatient admission, or administration via nasogastric tube—approaches associated with increased cost and resource use (4–6).

Taste aversion as a determinant of bowel preparation quality is well recognized, and multiple strategies have been developed to improve tolerability, including formulation changes, lower-volume regimens, flavoring, and adjunctive measures (7). However, these approaches primarily seek to improve the preparation itself. In contrast, the present manuscript proposes a conceptually distinct and underexplored approach: direct, transient modulation of gustatory perception as a potential complementary strategy in highly selected patients with severe taste intolerance.

## A serendipitous bedside observation

During admission for bowel preparation, a 65-year-old woman with repeated failed outpatient colonoscopy attempts was scheduled to receive laxatives via a nasogastric tube as an inpatient. As per routine practice, topical lidocaine spray (10% solution) was administered to the oropharynx to facilitate tube placement. Despite multiple applications, the tube was not tolerated. During a pause in the procedure, the patient spontaneously remarked that she “could not feel anything” in her mouth. Prompted by this observation, she was offered a small sip of the bowel preparation solution, which she tolerated with minimal aversion, no nausea, and no coughing. Encouraged by this response, additional lidocaine was applied, this time deliberately targeting the tongue surface rather than deeper pharyngeal structures, for a total of approximately 30 sprays (≈300 mg), noting that much of the initial dose had been expectorated. The patient subsequently tolerated oral ingestion of a substantial portion of the preparation. Colonoscopy the following day demonstrated excellent bowel cleansing.

This sequence of events was entirely unplanned. Lidocaine was not administered with the intent of modifying taste perception, nor was this effect anticipated. Nevertheless, the observation raises an intriguing question: could transient suppression of taste perception facilitate bowel preparation in patients otherwise unable to tolerate it?

## A sensory and anatomical perspective

Taste perception is not uniform across the tongue. Oropharyngeal application of topical anesthetic, as used for nasogastric tube placement, naturally reaches the posterior tongue where bitter taste perception predominates, while additional anterior tongue exposure further attenuates overall taste sensation (8). This distribution may explain the marked reduction in unpleasant taste without requiring deep pharyngeal anesthesia. Importantly, the intent is not to suppress the gag reflex, but to transiently blunt taste perception while preserving protective airway reflexes.

## Addressing aspiration risk and dosing

Any discussion of topical lidocaine in the context of oral intake appropriately raises concerns regarding aspiration (9). However, when applied primarily to the tongue for taste suppression rather than to the posterior pharynx, this risk is likely limited. Experimental data demonstrate that lidocaine gargling increases taste perception thresholds, particularly for bitterness, consistent with the reduction in unpleasant taste observed in this case (10). Notably, British Society of Gastroenterology (BSG) guidelines report no evidence that the combination of topical throat spray and sedation increases aspiration risk during upper gastrointestinal endoscopy, where lidocaine is routinely used with broader pharyngeal exposure than would be required for taste modulation alone (11). While the BSG guidance addresses aspiration risk in the context of upper gastrointestinal endoscopy with topical pharyngeal anesthesia, where exposure is typically limited to small volumes such as saliva, the present concept involves substantially larger fluid intake. Although the proposed approach emphasizes primarily lingual application, which may theoretically reduce pharyngeal exposure, this distinction should be interpreted with caution given the difference in volumes involved.

Another theoretical consideration is the effect of topical lidocaine on swallowing dynamics. Oropharyngeal application has been associated with increased inter-swallow intervals and reduced swallowing speed (12). While the present approach is primarily tongue-focused, a proportion of the applied lidocaine may reach the oropharynx, potentially leading to similar effects. This may, in some cases, counterbalance the potential benefits of taste suppression and warrants further evaluation.

Although concerns may arise regarding dosing, local prescribing guidance for airway and endoscopic procedures, such as the Swedish drug index (FASS), permits cumulative adult doses of approximately 400–600 mg (13). It should be noted that topical application in this context is associated with variable mucosal absorption, and a substantial proportion of administered lidocaine may be expectorated, suggesting that nominal dose may overestimate systemic exposure. Simple precautions, such as confirmation of intact swallowing, dose limitation, and brief supervision during ingestion, may further mitigate risk.

## Positioning within existing strategies

Existing approaches to improve bowel preparation tolerance include lower-volume preparations, split dosing, flavor modification, and adjunctive antiemetic strategies (14, 15). While these approaches aim to improve acceptability and adherence, they do not directly target gustatory perception itself. The present hypothesis should therefore be viewed not as a replacement for established strategies, but as a potential complementary approach for a narrowly defined subgroup of patients in whom conventional measures remain insufficient.

## Proposed framework for evaluation

This hypothesis could be evaluated in a pilot feasibility study involving patients with prior bowel preparation failure attributed to severe intolerance. A supervised, tongue-focused topical lidocaine intervention could be compared with standard care, with outcomes including validated tolerability measures, completion of preparation, and objective bowel cleansing scores such as the Boston Bowel Preparation Scale. Safety endpoints would include monitoring for swallowing impairment, coughing, and aspiration, alongside adherence to established dosing limits. Such an approach would provide initial evidence regarding feasibility, safety, and potential efficacy.

## From bedside observation to broader implications

This manuscript does not endorse the routine use of topical lidocaine to facilitate bowel preparation, nor does a single observation establish efficacy. Rather, it proposes a testable hypothesis: that supervised, tongue-focused modulation of taste perception may improve tolerance of bowel preparation in a small subset of patients with severe taste aversion. In the context of persistent preparation failure despite advances in formulation and scheduling, this perspective aligns with calls for more individualized approaches to bowel preparation.

Many advances in endoscopy arise from unexpected bedside observations rather than planned experimentation. Taste aversion remains an under-addressed barrier to adequate bowel preparation, and direct modulation of taste perception may represent a conceptually distinct approach that warrants systematic evaluation.

## Data Availability

The original contributions presented in the study are included in the article/supplementary material, further inquiries can be directed to the corresponding author.
